# Retroperitoneal Liposarcoma: Current Insights in Diagnosis and Treatment

**DOI:** 10.3389/fsurg.2015.00004

**Published:** 2015-02-10

**Authors:** Lucas E. Matthyssens, David Creytens, Wim P. Ceelen

**Affiliations:** ^1^Department of Surgery, Ghent University Hospital, Ghent, Belgium; ^2^Department of Pathology, Ghent University Hospital, Ghent, Belgium

**Keywords:** liposarcoma, sarcoma, surgery, radiotherapy, MDM2

## Abstract

Retroperitoneal liposarcoma (RLS) is a rare, biologically heterogeneous tumor that present considerable challenges due to its size and deep location. As a consequence, the majority of patients with high-grade RLS will develop locally recurrent disease following surgery, and this constitutes the cause of death in most patients. Here, we review current insights and controversies regarding histology, molecular biology, extent of surgery, (neo)adjuvant treatment, and systemic treatment including novel targeted agents in RLS.

## Introduction

### Anatomy of the retroperitoneum

The retroperitoneum (RP) forms together with the pre-peritoneum the extraperitoneal space. The RP space is an almost virtual and expandable space, defined anteriorly by the peritoneal extensions anchoring the transverse colon, the small bowel, as well as the ascending and descending colon, part of the duodenum, part of the pancreas, and a part of the liver (1, 2). The RP can be divided into perirenal and (anterior and posterior) pararenal spaces and contains several vital structures: the retroperitoneal organs (the pancreas, kidneys, adrenals, and part of the duodenum, ascending and descending colon), the greater abdominal vessels, the abdominal lymphatics, six major nerves and the autonomic (sympathetic) lumbar chains, and the connective tissue of fasciae, with the White line of Toldt as the fusion between the mesocolon and posterior RP (1).

Masses encountered in the RP can be benign, primary malignant, or metastatic. Primary retroperitoneal tumors (PRT) are those originating in the RP space, but not from the RP organs. Probably, the first report of a PRT was by Giovanni Battista Morgagni (1682–1771), describing in 1761, a retroperitoneal lipomatous tumor found at the autopsy of a 60-year-old woman (3). But it was Jean Fréderic Lobstein (1777–1835) of Strasbourg who launched the actual term “PRT” in his *Traité d’anatomie pathologique (1829)* (4). About three out of four PRTs are malignant (2, 5). PRTs are classified by their similarity with a certain type of mesenchymal tissue, with up to 2/3 being of mesodermal origin. Over 80% of mesodermal PRTs are malignant. Soft tissue sarcomas (STS), defined by James Stephen Ewing (1866–1943) in his book *Neoplastic Diseases (1919)* as “*unusual malignant tumors composed of cells of the mesodermal/connective tissue type*” (6), represent an extremely diverse group of more than 50 different types and subtypes of neoplasms, derived from adipose tissue, muscle, connective, vascular, or deep skin tissue and also bone and cartilage (2, 7–9). Although neural tissue is of (neuro-)ectodermal origin, malignant peripheral nerve sheath tumors (MPNST) are very often classified under STS as well.

### Natural history

Soft tissue sarcoma accounts for <1% of all malignant tumors in adults (2, 10), with an estimated incidence of 4–5/100,000/year in Europe (11). About 10–15% of adult STS are located in the RP (2, 12). Liposarcoma is the most common variant and accounts for 20% of all STS, and over 50% of RP sarcomas (13). Commonly classified on their histologic basis (14), STS are very heterogeneous and carry a varying prognosis. The natural behavior and outcome of STS are dependent of the age of the patient, anatomical site and depth, size, and resectability of the tumor, as well as of histology, grade, nodal disease, and distant metastasis (DM) (10). Most retroperitoneal soft tissue sarcomas (RPS), even of important size, rarely metastasize (2): only about 10% of RPS are found to have metastatic disease at presentation, which is mostly hematogenous and equally distributed to the lungs or the liver (2, 5). The presence of DM is an adverse prognostic factor for the outcome of all STS. In RPS, DM occurs in approximately 20–25% of patients and once DM is found, overall survival is poor, at a median of 13 months (10). DM in STS and RPS is largely dependent on the tumor’s malignancy grade (see below) (10). Because most RPS are low-grade, DM is rare, and the main problem is local control and recurrence. Their large size and deep location in an anatomically complex area containing a number of vital structures, makes the resectability of RPS difficult and sometimes impossible (5, 12). Patients who undergo complete (macroscopic) or even compartmental resection (R0 or R1) of the primary tumor have an improved prognosis with a 5-year overall survival of 54–70% (2, 15), yet 41–50% of these patients will demonstrate locally recurrent disease within 5 years after surgery (2, 5, 15). A review from the Memorial Sloan-Kettering Cancer Center (New York, NY, USA), the Royal Marsden Hospital (London, UK), and the French national multicenter study (10, 12, 13, 15) indicated that after more than 5 years, and even after complete macroscopic excision, local recurrence of RPS affects 60–70% of patients and is usually the cause of death (12).

### Predisposing factors

Most STS and especially RPS have no clearly identified cause (10). However, some predisposing factors have been identified: genetic alterations and exposure to radiation or chemical substances. The most important specific and non-specific genetic alterations predisposing to STS are listed in Table 1 (16). These genetically predisposed patients are even more at risk when exposed to ionizing radiation (17). In the general population, and especially in childhood (17–19), repeated computed tomography (CT)-scanning (18) and especially high dose ionizing radiation [as used in external beam radiation therapy (EBRT)] are associated with a higher risk of developing STS, with an estimated incidence of 5% after therapeutic radiation, e.g., for breast cancer, malignant lymphoma, and pediatric cancers (20, 21). The exact mechanism of STS development after EBRT remains, however, unknown (10). Most radiation-associated STS are high-grade/poorly differentiated and are found at the edge of the radiation field, with median latency periods of more than 8 years (range 6–20); mainly fibrosarcoma, osteogenic sarcoma, angiosarcoma, leiomyosarcoma, and undifferentiated pleomorphic sarcoma have been described after EBRT (10, 20). Toxic exposures to chemical agents have also lead to the development of STS, but this is at present mostly of historic interest and consisted mainly of exposure to phenoxyacetic acid/herbicides, thorium bromide/thorotrast, vinyl chloride, arsenic, asbestos, androgenic-anabolic steroids, dioxins, and chlorophenoles (20).

**Table 1:** **Predisposing genetic alterations for soft tissue sarcoma**.

Common name	Incidence	Gene mutation	Chrom.	Heredity	Sarcoma type	Reference
Neurofibromatosis type 1	1/2.000–4.000	NF1	17q11.2	Autos. Dom.	MPNST	(22)
Li-Fraumeni syndrome	1/5.000–20.000	TP53, hCHK2	17p13.1	Autos. Dom.	RMS, FS, UPS, OS, LPS, LMS a.o.	
FAP/Gardner syndrome	1/8.300–13.000	APC, MYH	5q22.2	Autos. Dom.	Desmoids (16% of pts)	(23)
Beckwith–Wiedemann-syndrome	1/13.700	NSD1, CDKN1C, H19	11p15, 5q35	Autos. Dom.	aRMS, eRMS	
Hereditary retinoblastoma	1/15.000–23.000	RB1	13q14	Autos. Dom.	Various STS	
Werner syndrome	3/million	WRN	8p12	Autos. Rec.	Various STS	(24)
Costello syndrome	115 pts in 2003	HRAS	11p15.5	Autos. Dom.	eRMS in 10/103	(25)
Nijmegen breakage syndrome	Unknown	NBS1	8q21.3	Autos. Rec.	RMS	

*Chrom., chromosome; Autos. Dom., autosomal dominant inheritance; Autos. Rec., autosomal recessive inheritance; MPNST, malignant peripheral nerve sheath tumor; RMS, rhabdomyosarcoma; aRMS, alveolar rhabdomyosarcoma; eRMS, embryonal rhabdomyosarcoma; pts, patients; FAP, familial adenomatous polyposis [modified from Ref. (16)]*.

## Clinical Presentation

Patients presenting with RPS are usually in their mid-fifties (median age 56 years) (5, 12, 13, 15, 21, 26–36), but RPS have been described to occur at all ages (2–98 years) (2, 5, 13, 15, 21, 26, 28, 31–33, 35–40). The gender distribution is supposedly equal (10, 12, 21), although some large retrospective series suggest a small surplus of female patients (mean 1.26M:1F) (2, 5, 13, 15, 26, 31–35, 37, 39, 40). Because the RP is a deep, expandable space without many bony boundaries, slowly growing tumors generally do not quickly cause signs or symptoms and may therefore grow to an important size before being discovered by increased abdominal girth, a palpable lump, or because of compression (causing gastrointestinal, urologic, or neurological symptoms). The majority (>75%) of PRT and RPS present “late,” with an important size. RPS is probably the largest tumors found in the human body (2). In fact, RPS measuring <5 cm is considered rare (35). RPS generally measure >5 cm, and mostly >10 cm diameter at presentation (34, 36). In the largest series of prospectively followed RPS, Lewis et al. found 94% of these tumors exceeding 5 cm in diameter and 60% exceeding 10 cm (2, 5). About 20–50% of RPS even exceed 20 cm in diameter at the time of resection (2, 15, 26, 30–33, 40, 41). Although probably decreasing with time [because of more widespread use of CT and magnetic resonance imaging (MRI)] between 60% (2, 39) and 80% (5, 12) of patients are believed to present with a palpable abdominal mass, and half of the patients have “pain” at presentation (21).

## Diagnostic Workup

The diagnosis and treatment of STS mandates a multidisciplinary approach ideally carried out in reference centers treating a high number of patients annually (11). Different imaging studies can be used in the evaluation of PRT/RPS: conventional radiographies of the abdomen usually indicate displacement of bowel and altered intestinal aeration, and may show signs of calcification in the tumoral mass (suggestive of teratoma). Ultrasound is useful as a quick first evaluator of abdominal complaints, but is of limited value for in-depth evaluation of RP masses, especially in adults with increased abdominal girth/obesity. Doppler/duplex ultrasound may offer additional information on the patency of the femoral and iliac vessels and of the inferior caval vein (ICV), especially in case of suspicion of partial or complete deep venous thrombosis due to vascular compression. The diagnostic investigation of choice to evaluate PRT/RPS is contrast-enhanced CT-scanning or MRI of the abdomen and pelvis (11, 12, 21, 42). They will determine the anatomical location of the tumor, its size, and probable origin, the relationship of the tumor to adjacent visceral and neurovascular structures, possible compression or invasion, and the presence or absence of transperitoneal spread or liver or lung metastases (12, 43). Liposarcomas demonstrate a characteristic appearance on CT and MRI with a predominantly fatty component (12). MRI does not cause added radiation exposure and may be specifically required under certain circumstances (12), e.g., in pediatric patients, in cases of myxoid or round cell liposarcoma (MRI of the spine, because of the higher risk for spinal metastasis compared to other STS), or in alveolar soft part sarcomas and angiosarcomas (MRI of the brain, for their propensity to metastasize to the brain especially in the presence of pulmonary metastasis) (44). MRI cannot, however, reliably distinguish between benign and malignant tissue, but diffusion-weighted MRI (DW-MRI) seems a very promising technique and is under study (12, 43, 45).

The “staging” investigation of choice for the detection of DM is contrast-enhanced CT-scan of the chest and abdomen (12). ^18^F-Fluorodeoxyglucose (FDG)-positron emission tomography (PET) – CT scan may provide additional functional/biological information about the retroperitoneal tumor and may possibly differentiate a high-grade from a low-grade STS (46). Apart from grading, FDG-PET may also aid in staging (detection of metastases), restaging and in the evaluation of treatment response and follow-up, by detecting residual masses or recurrences after attempted radical surgery for STS. In a recent study of 102 STS at UCLA, the tumor glycolytic phenotype correlated significantly with the histologic grade in 91% of tumors, which may offer prognostic significance – although FDG-PET could not reliably distinguish among French Fédération Nationale des Centers de Lutte contre le Cancer (FNCLCC)-grade 2 and grade 3 STS and the various subtypes. When regarding the liposarcomas studied, in 6 of 16 (38%), there was a SUVmax <2.5 g/mL, suggesting that for this STS-subtype, FDG-PET-based treatment monitoring might be difficult. Further prospective studies on the value of PET for STS are underway (44).

## Classification, Staging, and Grading

The distribution of sarcoma subtypes in the RP differs from other localizations, with a predominant role (75–85%) for liposarcoma and leiomyosarcoma. RPS is classified using the *World Health Organization (WHO) Classification of soft tissue tumors* (Table 2) (14). Based on its histologic type and subtype, the tumor is classified into one of four categories: benign, intermediate (locally aggressive), intermediate (rarely metastasing), and malignant (14).

**Table 2:** **WHO classification of soft tissue tumors of intermediate malignant potential and malignant soft tissue tumors**.

Adipocytic tumors
Intermediate (locally aggressive)
Atypical lipomatous tumor/well-differentiated liposarcoma
Malignant
Dedifferentiated liposarcoma
Myxoid/round cell liposarcoma
Pleomorphic liposarcoma
Mixed-type liposarcoma
Liposarcoma, not otherwise specified
Fibroblastic/myofibroblastic tumors
Intermediate (locally aggressive)
Superficial fibromatoses (palmar/plantar)
Desmoid-type fibromatoses
Lipofibromatosis
Intermediate (rarely metastasizing)
Solitary fibrous tumor and hemangiopericytoma (including lipomatous hemangiopericytoma)
Inflammatory myofibroblastic tumor
Low-grade myofibroblastic sarcoma
Myxoinflammatory fibroblastic sarcoma
Infantile fibrosarcoma
Malignant
Adult fibrosarcoma
Myxofibrosarcoma
Low-grade fibromyxoid sarcoma/hyalinizing spindle cell tumor
Sclerosing epithelioid fibrosarcoma
So-called fibrohistiocytic tumors
Intermediate (rarely metastasizing)
Plexiform fibrohistiocytic tumor
Giant cell tumor of soft tissues
Malignant
Pleomorphic malignant fibrous histiocytoma (MFH)/undifferentiated pleomorphic sarcoma
Giant cell MFH/undifferentiated pleomorphic sarcoma with giant cells
Inflammatory MFH/undifferentiated pleomorphic sarcoma with prominent inflammation
Smooth muscle tumors
Malignant
Leiomyosarcoma
Skeletal muscle tumors
Malignant
Embryonal rhabdomyosarcoma (including spindle cell, botryoid, anaplastic)
Alveolar rhabdomyosarcoma (including solid, anaplastic)
Pleomorphic rhabdomyosarcoma
Vascular tumors
Intermediate (locally aggressive)
Kaposiform hemangioendothelioma^a^
Intermediate (rarely metastasizing)
Retiform hemangioendothelioma
Papillary intralymphatic angioendothelioma
Composite hemangioendothelioma
Malignant
Epithelioid hemangioendothelioma
Angiosarcoma of soft tissue
Tumors of peripheral nerves
Malignant
Malignant peripheral nerve sheath tumor
Epithelioid malignant peripheral nerve sheath tumor
Chondro-osseous tumors
Malignant
Mesenchymal chondrosarcoma
Extraskeletal osteosarcoma
Tumors of uncertain differentiation
Intermediate (rarely metastasizing)
Angiomatoid fibrous histiocytoma
Ossifying fibromyxoid tumor (including atypical/malignant)
Mixed tumor/myoepithelioma/parachordoma
Malignant
Synovial sarcoma
Epithelioid sarcoma
Alveolar soft part sarcoma
Clear cell sarcoma of soft tissue
Extraskeletal myxoid chondrosarcoma (“chordoid” type)
Primitive neuroectodermal tumor (PNET)/extraskeletal Ewing tumor
Peripheral primitive neuroectodermal tumor (pPNET)
Extraskeletal Ewing tumor
Desmoplastic small round cell tumor
Extra-renal rhabdoid tumor
Malignant mesenchymoma
Neoplasms with perivascular epithelioid cell differentiation (PEComa)
Clear cell myomelanocytic tumor
Intimal sarcoma

*^a^Since the last edition of the WHO classification, two cases of well-documented regional metastasis of kaposiform hemangioendothelioma have been reported (47) raising the issue of whether or not kaposiform hemangioendothelioma might be more appropriately included in the category of “intermediate (rarely metastasizing)” instead of “intermediate (locally aggressive).” This will undoubtedly be addressed in the next WHO classification of tumors of soft tissue*.

Different staging systems have been in use for predicting the systemic outcomes of patients with STS, but a specific staging system for RPS is not (yet) available. The revised *UICC/AJCC-7 cancer staging system for the prognostic classification of sarcomas* is the most commonly used; since 1977 this includes the histologic grade (Table 3) (48). Other staging systems include the “*surgical staging system*” (SSS) by Enneking and the Musculoskeletal Tumor Society (49) and the postsurgical classification system by the Sarcoma Disease Management Team at Memorial Sloan-Kettering Cancer Center (MSK-system) (50).

**Table 3:** **Sarcoma staging system, seventh edition of the American Joint Committee on Cancer/International Union against Cancer (UICC/AJCC-7, 2010)**.

**T: primary tumor**				
Tx	Primary tumor cannot be assessed			
T0	No evidence of primary tumor			
T1	Tumor ≤5 cm			
	T1a Superficial tumor (above the non-invaded fascia			
	T1b Deep tumor (under the fascia or with invasion of the fascia)			
T2	Tumor >5 cm			
	T2a Superficial tumor			
	T2b Deep tumor (retroperitoneum = always deep)			
**N: regional lymph nodes**				
Nx	Lymph node status unknown			
N0	No regional lymph nodes			
N1	Regional lymph node metastasis			
**M: distant metastasis**				
Mx	Distant metastasis unknown
M0	No distant metastasis
M1	Distant metastasis
**G: histopathological grading**				
TNM-two grade system	Three grade systems	Four grade systems	
		Gx	Grade cannot be assessed	
Low grade	Grade 1	G1	Well-differentiated	
		G2	Moderately differentiated	
High grade	Grade 2	G3	Poorly differentiated	
	Grade 3	G4	Undifferentiated	
**Stage grouping**				
Stage Ia	T1a	N0	M0	Low grade
	T1b	N0	M0	Low grade
Stage Ib	T2a	N0	M0	Low grade
	T2b	N0	M0	Low grade
Stage IIa	T1a	N0	M0	High grade
	T1b	N0	M0	High grade
Stage Iib	T2a	N0	M0	High grade
Stage III	T2b	N0	M0	High grade
Stage IV	Any T	N1	M0	Any grade
	Any T	Any N	M1	Any grade

*Remark: for bone and soft tissue sarcoma, preference is given to a 2-tier instead of 3- or 4-tier system: low versus high grade*.

Histologic grade represents the most important indicator of metastatic risk and OS in adult STS. The main objective of grading is to select patients for adjuvant chemotherapy (51). The concept of histological grade in sarcoma was introduced by Broders in 1920 and since then, various 2-, 3-, or 4-tier grading systems have been in use. There is at present no single generally agreed upon grading system for STS. Since the 1980s, the FNCLCC (52, 53) and the US National Cancer Institute (NCI) (54) systems are the most commonly used grading systems for STS (Table 4). Both are 3-grade systems based on histologic tumor differentiation, mitotic rate/activity, and percentage of tumor necrosis. The NCI system also requires quantification of cellularity and pleomorphism for certain sarcoma subtypes, which is difficult to determine objectively. The UICC/AJCC-7 STS staging system is, however, not fully adapted for RPS. As the majority of RPS are large and deeply situated, the prognostic value of “T” (size and depth) is less applicable (all are T2b) and the same is true for “N,” as most RPS do not develop lymphatic metastasis. The system is not applicable for local recurrences (very common in RPS) and grading remains difficult with demonstrable interobserver discordances, even among experienced pathologists. Therefore, the surgical oncology team from MD Anderson Cancer Center (Houston, TX, USA) proposed in 2009 a novel practical “histology-based prognostic system” to predict overall survival in all RPS patients (34). This system stratifies RPS patients into three risk groups according to tumor histology, with “Atypical lipomatous tumor” (ALT, well-differentiated liposarcoma) having the best prognosis, “non-ALT liposarcoma” (non-ALT LPS) having the worst overall survival and “Other” histology (non-LPS) having an intermediate prognosis. This system is also applicable for recurrent disease, and further risk stratification can still be determined within each of the groups (34).

**Table 4:** **The French Fédération Nationale des Centers de Lutte Contre le Cancer (FNCLCC) Grading System**.

**Tumor differentiation**	
Score 1 Sarcomas that closely resemble normal adult mesenchymal tissues	
Score 2 Sarcomas for which histologic typing is certain	
Score 3 Embryonal and undifferentiated sarcomas, synovial sarcoma, and sarcomas of uncertain differentiation	
**Mitotic count**	
Score 1 0–9 mitoses/10 hpf
Score 2 10–19 mitoses/10 hpf
Score 3 ≥20 mitoses/10 hpf
**Tumor necrosis**	
Score 0 No necrosis
Score 1 <50% tumor necrosis
Score 2 ≥50% tumor necrosis
Histologic grade (tumor differentiation + mitotic count + tumor necrosis)	
Grade 1 (low grade)	Total score: 2 or 3
Grade 2 (intermediate grade)	Total score: 4 or 5
Grade 3 (high grade)	Total score: 6, 7, or 8

*FNCLCC, Fédération Nationale des Centres de Lutte Contre le Cancer; hpf, high-power field*.

*Data from Ref. (52)*.

On the other hand, as for other neoplasms, *molecular markers* hold also great promise for refining our ability to establish early prognosis and to predict response to treatment in STS/RPS (“molecular grading”) (51). Molecular profiling analysis by microarray technology has been performed in STS and a 67-gene expression signature called CINSARC has recently been identified as a clinically applicable prognostic marker (51). However, the value of CINSARC for predicting the response to treatment is not yet known and will soon be validated in prospective independent series (51). Since almost a decade, *cancer nomograms* have been developed, mainly instigated by the Sarcoma Disease Management Team of MSKCC. Nomograms are being increasingly accepted to predict risk of recurrence and disease-specific death, and to aid the clinician in counseling patients and planning for surveillance and follow-up (13). Nomograms for RPS and liposarcoma of the RP have recently been published by different teams, based on the histologic subtype, margin of resection, contiguous organ resection, and age as prognostic markers for survival.

## Histology and Molecular Biology

**Figure 1:**
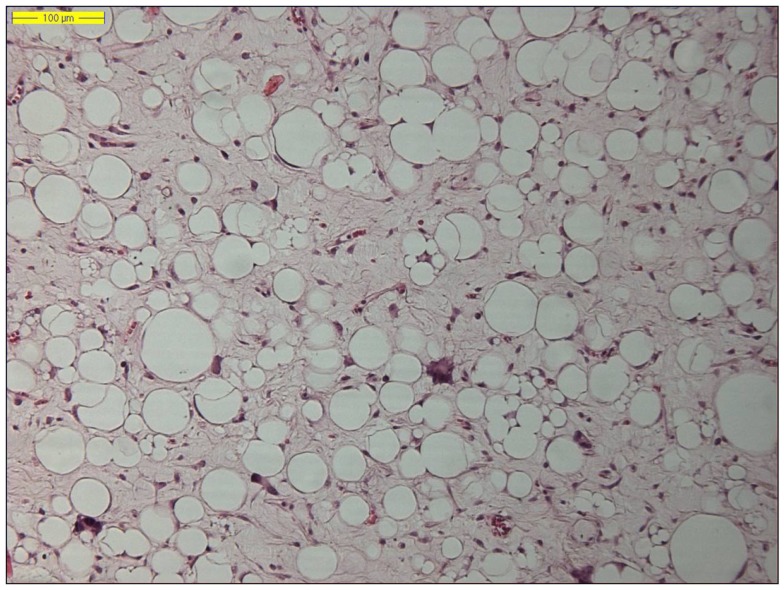
**Histology of a lipoma-like well-differentiated liposarcoma (hematoxylin and eosin, original magnification 200×)**.

**Figure 2:**
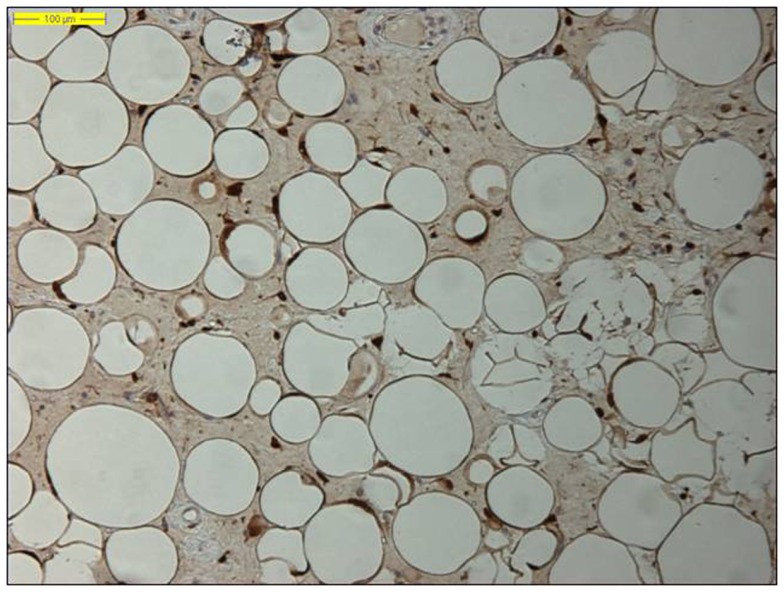
**Nuclear MDM2 immunohistochemical overexpression in the atypical adipocytes in a lipoma-like well-differentiated liposarcoma (original magnification 200×)**.

**Figure 3:**
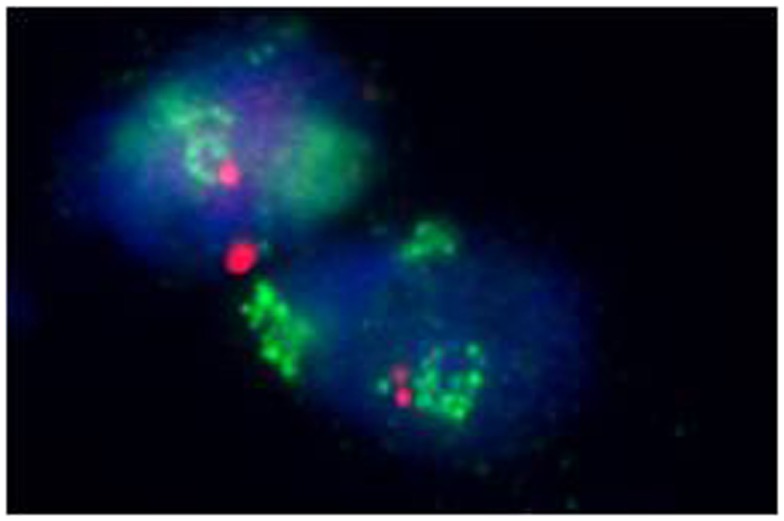
**Amplification of the MDM2 gene in a well-differentiated liposarcoma (fluorescence *in situ* hybridization, FISH)**.

**Figure 4:**
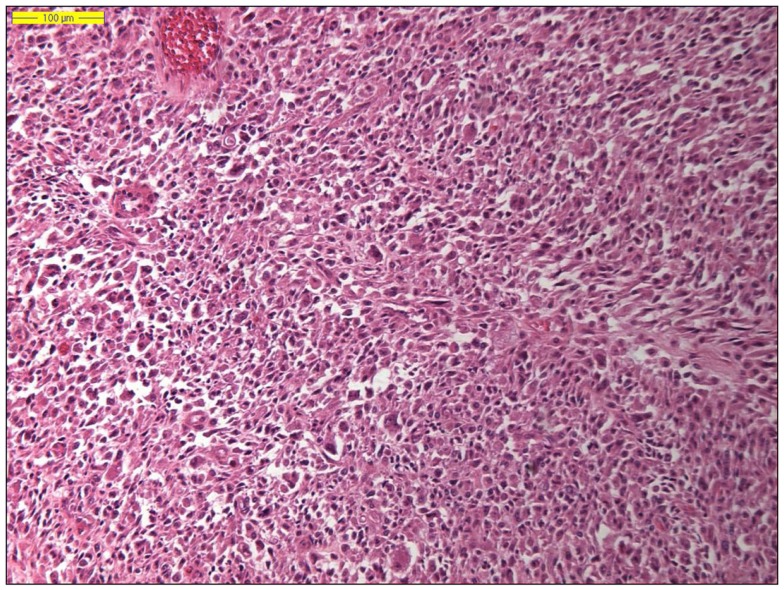
**Histology of a dedifferentiated liposarcoma (hematoxylin and eosin, original magnification 100×)**.

The histological classification of liposarcoma has evolved significantly over past several decades, in large part owing to the advances in our understanding of its molecular genetics. The recently updated World Health Organization (WHO) classification of soft tissue and bone tumors recognizes four major liposarcoma subtypes: atypical lipomatous tumor/well-differentiated liposarcoma [which includes the adipocytic (or lipoma-like), sclerosing, inflammatory and spindle cell variants], dedifferentiated liposarcoma, myxoid liposarcoma, and pleomorphic liposarcoma (Figures 1–4) (55, 56). These four main subgroups are characterized by distinctive morphologies, as well as unique genetic findings. A fifth subtype (the so-called “mixed or combined liposarcoma”), which was still a separate entity in the 2002 WHO classification, has been removed from the most recent 2013 WHO classification, based on the consensus view that those rare cases probably represent examples of (variants of) dedifferentiated liposarcoma. It is important to emphasize that atypical lipomatous tumor and well-differentiated liposarcoma are synonyms, describing lesions, which are identical both morphologically and karyotypically. Use of the term atypical lipomatous tumor is determined principally by tumor location and resectability. In sites such as the RP, it is usually impossible to obtain a wide tumor free surgical excision margin of more than 2 cm. In such cases, local recurrence is common and often leads to death, even in the absence of dedifferentiation or metastasis. At these sites, thus, the term well-differentiated liposarcoma is used rather than atypical lipomatous tumor (55, 56). Histopathology is the gold standard in the diagnostic traject of lipomatous tumors. In addition to tumor size and anatomic location, one of the most important determining factors for the prognosis of liposarcoma patients is the histological liposarcoma subtype, further underlining the importance of correct subclassification. However, establishing the correct lipomatous tumor subtype can be laborious and requires in some instances a histological assessment together with immunohistochemistry and molecular analyses using fluorescence *in situ* hybridization (FISH), polymerase chain reaction (PCR), multiplex ligation-dependent probe amplification (MLPA), and/or array comparative genomic hybridization (aCGH). Finally, with a growing number of molecularly targeted agents in oncology, molecular testing will become increasingly important in guiding treatment strategies of liposarcomas in the near future.

The two by far most common (lipo)sarcoma subtypes in the RP are the well-differentiated liposarcoma (Figure 1) and dedifferentiated liposarcoma, followed by the leiomyosarcoma. Primary myxoid liposarcomas, occurring predominantly in the lower limbs of young to middle-aged adults, are extremely rare and may be “non-existing” in the RP. Therefore, a diagnosis of primary retroperitoneal myxoid liposarcoma should be regarded with suspicion, as most such cases represent either metastatic myxoid liposarcoma or well-differentiated/dedifferentiated liposarcoma with myxoid stromal change (57, 58). Pleomorphic liposarcoma, defined as a high-grade pleomorphic sarcoma showing variable amounts of lipoblastic differentiation, arise most often in the limbs of elderly patients and are extremely rare in retroperitoneal location (59, 60).

Well-differentiated liposarcoma is a genetically distinct group of lesions. With the exception of the spindle cell variant, all well-differentiated liposarcoma subtypes share the same genetic aberration and are characterized by supernumerary ring and/or giant rod chromosomes containing amplified segments from the 12q13-15 region where several proto-oncogenes including murine double minute type 2 (*MDM2*), cyclin-dependent kinase 4 (*CDK4*), high-mobility AT-hook 2 (*HMGA2*), and tetraspanin 31 (*TSPAN31* or *SAS*) are located (61–69). *MDM2* is the most frequent amplified gene, close to 100%, and *CDK4* is shown to be amplified in over 90% of cases (70, 71). Co-amplification of *MDM2* and *CDK4* is a common feature in well-differentiated liposarcoma and is thought to be the initiating “driving” factor in fat tumorigenesis, resulting in proliferation through combined effects upon *p53* (by inactivating TP53) and the cell cycle (by *RB1* phosphorylation), respectively. It has been suggested that *CDK4* provides a selection advantage in well-differentiated liposarcoma and may contribute to transformation as *CDK4* negative well-differentiated liposarcoma exhibit more favorable prognostic features (64, 70–72). Amplification of *MDM2*, *CDK4*, and H*MGA2* can be detected by molecular techniques including FISH, PCR, MLPA, or aCGH techniques (68, 73–75). P53 mutations are rarely seen in well-differentiated and dedifferentiated liposarcomas, but are commonly in pleomorphic liposarcomas.

Barretina et al. showed 16.7% of pleomorphic liposarcoma cases had mutations in p53 (76). Similarly, high p53 mutations rates (approximately 60%) were observed in pleomorphic liposarcoma by Ghadimi et al. (77).

Moreover, identifying *MDM2* amplification, as well as overexpression of the corresponding MDM2 protein by immunohistochemistry, has proved an adjunctive tool in the diagnosis of lipomatous neoplasms, especially in the diagnosis of a well-differentiated liposarcoma, because *MDM2* amplification is absent in “ordinary” lipomas (Figures 2 and 3) (78–81). Molecular testing should be considered for “relapsing lipomas,” tumors with questionable cytologic atypia (pathologists tend to overestimate the degree of cytologic atypia), or for large lipomatous tumors (>15 cm) without diagnostic cytologic atypia (81).

**Figure 5:**
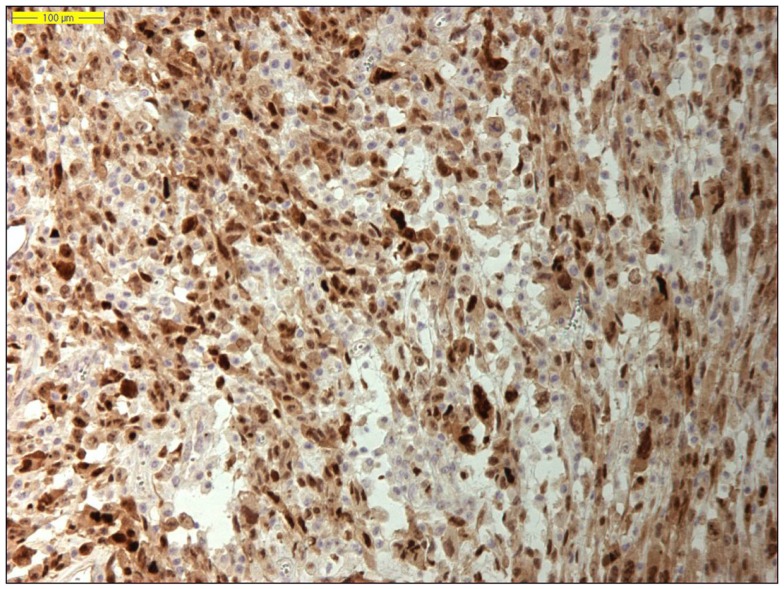
**Nuclear MDM2 immunohistochemical overexpression in dedifferentiated liposarcoma (original magnification 200×)**.

**Figure 6:**
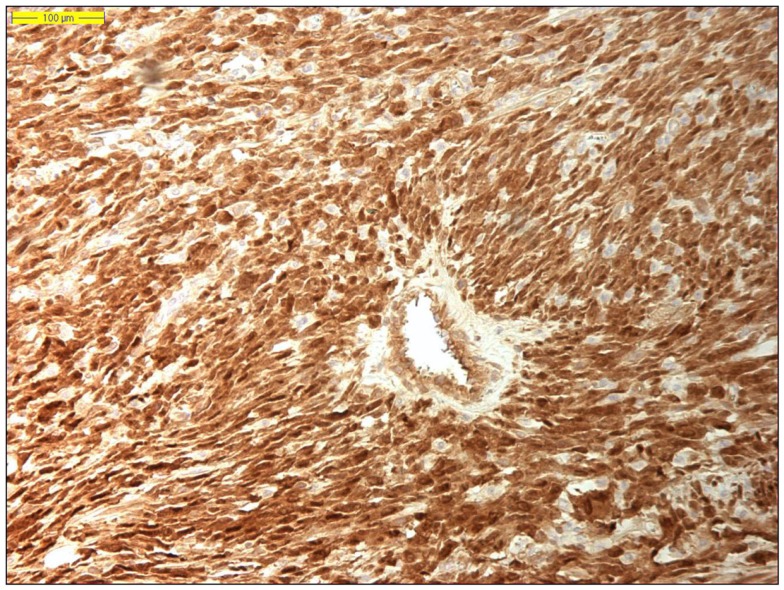
**Nuclear CDK4 immunohistochemical overexpression in dedifferentiated liposarcoma (original magnification 200×)**.

Dedifferentiated liposarcoma is a biologically fascinating lesion, in which morphology, genetics, and clinical behavior converge to define a distinctive clinicopathological entity. The term “tumor dedifferentiation” as established in 1971 by Dahlin and Beabout, characterizes “the morphological progression of a low-grade tumor to a less differentiated neoplasm with a more aggressive behavior” (82). Dedifferentiated liposarcoma is traditionally defined as “a non-lipogenic high-grade sarcoma arising from a well-differentiated liposarcoma that confers metastatic potential.” The term dedifferentiated liposarcoma was first introduced by Evans in 1979, describing a liposarcoma containing a well-differentiated liposarcoma component juxtaposed to areas of high-grade non-lipogenic sarcoma and was believed to occur from well-differentiated liposarcoma after several years (83). The RP is the most common location, outnumbering somatic soft tissue by at least 5/1 (55, 56, 84). More than 90% of dedifferentiated liposarcoma arises *de novo* (synchronous), while <10% occurs in recurrences (metachronous). Dedifferentiated areas in dedifferentiated liposarcoma exhibit a wide morphological spectrum (84). Histologically, most cases of dedifferentiated liposarcoma show areas of high-grade poorly differentiated sarcoma resembling high-grade myxofibrosarcoma, fibrosarcoma, malignant solitary fibrous tumor, or pleomorphic sarcoma NOS (Figure 4). In about 5–10% of cases, the dedifferentiated component shows divergent differentiation featuring myogenic, angiosarcomatous, or osteochondromatous components (85–88). Several recent studies have reported that most sarcomas diagnosed as poorly differentiated sarcomas and arising in the RP are, in fact, dedifferentiated liposarcomas and can now be diagnosed as such on the basis of *MDM2* amplification even in challenging cases of a non-lipogenic undifferentiated sarcoma without an atypical adipocytic component (89–91). Like atypical lipomatous tumor/well-differentiated liposarcoma, dedifferentiated liposarcoma is characterized by presence of supernumary ring and/or giant rod chromosomes containing amplified segments from the 12q13-15 region (55, 56, 62, 63, 65, 66, 84). Intensive research has identified several oncogenes residing in this region, including *MDM2*, *CDK4*, *HMGA2*, *TSPAN31* (*SAS*), *YEATS4*, *miR-26a-2*, *CPM*, *OS1*, *OS9*, *CHOP* (*DDIT3*), and *GLI1* (63). The most evidence, to date, demonstrates an oncogenic role in dedifferentiated liposarcoma, like the atypical lipomatous tumor/well-differentiated liposarcoma, for *MDM2*, *CDK4*, *HMGA2*, and *TSPAN31* (*SAS*) (55, 64, 70, 71, 84). Wang et al. described consistent amplification of the fibroblast growth factor receptor substrate 2 gene (*FRS2*) in dedifferentiated (and well-differentiated) liposarcoma (92). Recently, *STAT6* (12q13) amplification and overexpression was described in a subset of dedifferentiated liposarcoma, further underlining the genomic complexity and heterogeneity of ring and giant marker chromosomes of this tumor type, particularly concerning amplicons originating from the chromosomal region 12q13-15 (93, 94). Despite its typically high-grade morphology, dedifferentiated liposarcoma is much less aggressive than other types of high-grade pleomorphic sarcoma (55, 56, 95, 96). Dedifferentiation is associated with a 15–20% metastatic rate; however, mortality is related more often to uncontrolled local recurrences than to metastatic spread. Therefore, it is of clinical importance to distinguish a dedifferentiated liposarcoma from a *de novo* high-grade pleomorphic sarcoma of some other type (97). A recent study by Thway et al. have suggested that the immunohistochemical trio of CDK4, MDM2, and the cell cycle regulator p16 is an useful ancillary diagnostic tool distinguishing dedifferentiated liposarcomas from pleomorphic and myxoid liposarcomas (Figures 5 and 6) (98).

The concept of dedifferentiation in liposarcoma has undergone an evolution in the last several years and the traditional views have been modified by the concept of low-grade dedifferentiation in dedifferentiated liposarcoma. Where it was once assumed that all dedifferentiated tumors manifested themselves as high-grade, undifferentiated sarcoma-like lesions, the concept of low-grade dedifferentiation has increasingly been recognized, with areas resembling low-grade myxofibrosarcoma, desmoid fibromatosis, well-differentiated fibrosarcoma, and even dermatofibrosarcoma protuberans (55, 56, 84). The significance of this lower grade of progression is, to date, not completely known and is still controversial. However, there is some suggestion that the lower grade progression carries a better prognosis than the high-grade undifferentiated type of dedifferentiated liposarcoma (55).

The mechanisms responsible for progression from well-differentiated liposarcoma to dedifferentiated liposarcoma are incompletely understood. Since *MDM2* and *CDK4* amplifications are present in both well-differentiated and dedifferentiated liposarcoma, the presence of these amplifications as such are not triggers for dedifferentiation in liposarcomas. As a group, dedifferentiated liposarcomas show more complex chromosomal aberrations than do well-differentiated liposarcomas. Chromosomal imbalances additionally to the 12q13-q15 amplicon, including amplifications in 1p32 (including *JUN*), 1q21-q24, and/or 6q23 (including the *ASK1* or *MAP3K5* gene), have been reported to be more frequent in dedifferentiated liposarcoma than in well-differentiated liposarcoma (69). Recent studies into the well-differentiated liposarcoma de-differentiation process have suggested a role for c-Jun N-terminal kinase (*JNK*) pathway (99). The proto-oncogene *c-Jun* encodes part of the activator protein transcription factor (AP-1) complex involved in cell proliferation, transformation, and apoptosis. *ASK1* activates *JNK* ultimately leading to *c-Jun* activation and peroxisome proliferator-activated receptors (PPAR) gamma inactivation. PPAR gamma is involved in the adipocytic differentiation process and its inhibition may result in dedifferentiation. Co-amplification of 1p32 and 6q23 that contain *c-Jun* and apoptosis signaling kinase 1 (*ASK1*) are seen in dedifferentiated liposarcoma but not in well-differentiated liposarcoma (84, 99–103).

## Treatment

### Surgery

Surgery is the mainstay of treatment of non-metastatic retroperitoneal liposarcoma (RLS). Whenever possible, macroscopically complete resection should be aimed at, often requiring en-bloc removal of adjacent structures such as the abdominal wall, psoas, or paravertebral muscles. In an attempt to optimize the surgical approach to these patients and provide a standardized, reproducible technique, technical guidelines were recently provided by E-Surge, a master class in sarcoma surgery, and the EORTC soft tissue and bone sarcoma group (104). Areas of uncertainty include the necessity of pretreatment biopsy, and the impact of surgical radicality versus disease biology on local control and long-term survival.

### Pretreatment biopsy

In most patients with RLS, the iconographic appearance (location, density, displacement rather than invasion of adjacent organs) is nearly diagnostic and pretreatment biopsy therefore unnecessary. As a consequence, it has been argued that pretreatment biopsy does not offer any value in patients with a resectable retroperitoneal mass (105). In some patients, however, radiology may suggest a different pathology that may not require surgery as the first approach (lymphoma, Ewing sarcoma, GIST). Also, in patients at risk for incomplete resection and in whom neoadjuvant radiotherapy is planned, pretreatment histological confirmation is mandatory. In these patients, image guided core or fine needle aspiration biopsy are reliable and safe, and preferred over open or laparoscopic approaches, which may be associated with a higher risk of tumor spillage and may compromise future surgical strategy by altering tissue planes (106–109).

### Extent of surgery versus tumor biology

**Figure 7:**
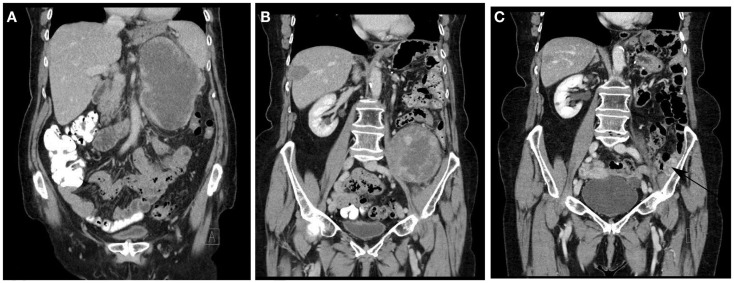
**Typical disease course of dedifferentiated liposarcoma in an elderly patient, who presented with a recurrent RLS in February 2011 2 years after primary surgery (A)**. The patient was treated with neoadjuvant radiotherapy (45 Gy in fractions of 1.8 Gy) and macroscopically completes resection. In May 2012, a solitary metastasis in the right liver lobe **(B)** was treated with RF ablation. In September 2012, a solitary 1.6 cm metastasis was removed thoracoscopically from the right lower lobe. In May 2013, a second retroperitoneal recurrence developed **(B)** for which repeat macroscopically complete surgery was performed. In September 2014, a small recurrence was noted against the left iliac bone [**(C)** arrow] for which additional surgery is planned. No other local or metastatic locations were noted.

In contrast to limb (lipo)sarcoma, removal of the entire tumor with a rim of normal tissue is usually precluded in (large) RLS due to adjacent large vessels, nerves, or bony structures. As a consequence, many patients develop locally recurrent disease in the abdomen, which constitutes the cause of death in approximately three out of four patients (Figure 7) (34). Several centers therefore advocate liberal compartmental, en-bloc resection of adjacent organs in order to reduce the risk of local relapse (30, 32). On the other hand, although all liposarcomas share the amplification of 12q13-15, resulting in overexpression of MDM2 and CDK4, the molecular biology of the disease is heterogeneous, and likely to be differing between limb and retroperitoneal disease locations, given the fact that recent molecular studies highlighted major ontogenetic differences between normal subcutaneous and visceral (including retroperitoneal) fat tissue (110). High grade, dedifferentiated tumors are at much higher risk to recur and spread systemically, and therefore, unlikely to benefit from extensive surgery (111).

The published surgical experience is entirely based on retrospective analyses and difficult to interpret due to significant heterogeneity in terms of care setting (monocentric versus multicentric or multinational), sarcoma type, stage distribution, type of presentation (primary versus recurrent disease), staging system used, and pathology methods used (Table 5). Nevertheless, some observations are rather consistent. First, as is clear from the results of multivariate analyses and the associated estimates of the hazard or risk ratio, advanced tumor grade represents the most important adverse prognostic factor for overall survival as well as for local recurrence. Second, macroscopically incomplete, piecemeal resection, or tumor rupture are associated with a dismal outcome and should not be attempted unless for symptomatic reasons. The efficacy of extended, liberal resection is equivocal, while some authors have identified multiorgan resection and microscopic margin status (R0 versus R1) as independent prognosticators for local recurrence; others found that only tumor biology (grade) and macroscopically complete resection were associated with outcome. Obviously, interpretation of these data is hampered by the fact that in these large tumors, precise determination of R0 resection is not a sinecure. When considering the potential benefit of extensive surgery, it should be noted that a significant proportion of patients will develop multifocal recurrence, including at sites remote from the primary tumor location. Tseng and coworkers found that as many of 50% of patients with recurrent RLS presented with multifocal disease; importantly, type or extent of surgery did not predict recurrence outside of the resection field on univariate logistic regression analysis (37). Although tumor spill or incomplete resection may explain multifocal recurrence, it has been suggested that a “field change” of the entire intra-abdominal fat tissue may underly the observation of remote (out-of-field) recurrence (112). Genomic analyses of normal retroperitoneal fat as well as tumor samples may provide further insight into this phenomenon.

**Table 5:** **Prognostic factors of local control and overall survival outcome in selected published series of surgically treated retroperitoneal (lipo)sarcoma**.

Author	*N*	LiSa%	CoRes%	5 years OS/DSS%	5 years DFS/LRFS%	Prognostic factors							
						Overall survival				Local recurrence/DFS			
						Univariate		Multivariate		Univariate		Multivariate	
							
						Significant	NS	Significant	NS	Significant	NS	Significant	NS
Kilkenny (113)	63	22	78	48	–	–	Multivisceral resection Gender, biopsy type Vascular involvement Adjuvant therapy Location, race	Compl. resection (<0.0001) Grade (0.001)	Metastatic dis Multiple resections Margin status Histol type	–	–	–	–

Lewis (5)	500	41	42	54	59	Grade Margin status	Gender, size Age, histol type	Grade (2.0–5.0) Size (1.1–2.7) Incomplete res (2.5–6.5)	–	Gender, grade Histol type	Age, size Margin status	Grade (1.2–3.4) Histol type (1.5–4.6)

Stoeckle (36)	165	26	65	46	42	RT, histol type Complete resection	–	No compl remission (1.6–5.1) Grade 3 (1.5–7.6) T3 stage (1.1–3.4)	Size, gender Location	–	–	No RT (1.8–6.3) Grade 3 (1.4–7.3)	T stage, size Chemotherapy Histol type

Ferrario (35)	130	41	95	65	–	–	–	Grade (0.001) Extent of resection (0.01)	Size	–	–	–	–

Gronchi (33)	167	57.5	88	53.6	27.6	–	–	Grade 3 (3.1–8.8) RT (0.4–0.9)	Tx period Size, chemotherapy	–	–	Tx period (0.4–0.9) Grade 3 (1.5–4.1) RT (0.4–1.01)	Size Chemotherapy

Van Dalen (38)	143	38	54	39	22	Age, histol type Grade, incomplete res Distant metastasis	Locoregional spread	Grade (1.2–4) Incomplete res (1.7–4.2)	–	–	–	Intermediate grade (1.3–4.9)	

Lehnert (21)^a^	110	53.6	67	49	40	Grade, margin status Blood loss Adjacent organ invasion	Primary vs recurrent Age, size	Grade (1.3–28.2) Blood loss (1.1–4.9)	Age, margin status Adjacent organ invasion Primary vs recurrent	Grade Primary vs recurrent Margin status Adjacent organ invasion	Size, age Blood loss	Grade (2.7–34.6) Prim vs rec (0.99–4.4)	Age, size Margin status Blood loss Adj organ invasion

Bonvalot (15)	382	50	73	57	51	Histol type, grade Tumor rupture Incomplete res Margin status	Gender, age, size RT # Organs resected	Grade 3 (2.03–6.3) Margin status (1.1–2.7) Tumor rupture (1.4–3.3)	Histol type	Grade 3, histol type No multiorgan res Margin status Tumor rupture <30 cases/center	–	Grade 3 (1.5–4.6) No multiorgan res (1.2–3.9) Margin status (1.2–2.9) Tumor rupture (1.5–3.6) # Cases/center

Strauss (26)	200	76	85	68.6	54.6	Grade, size ALT histol type Incomplete res	Age, weight R0 vs R1	Grade 3 (6.5–46.3) Incomplete res (1.5–5.8)	Size	Grade Incomplete res	Age, size Weight	Grade 3 (2.4–9) Incomplete re (2.3–5.9)	

Gronchi (29)	523	52.7	91	56.8	39.4	–	–	Age (1.04–1.7) Size (1.6–3.4) Grade 3 (9.2–77.9) Multifocality (1.4–4.02) Incomplete res (1.05–2.75)	Histol type	–	–	Size (1.2–2.2) Grade 3 (4.1–18.3) Multifocality (1.6–4.8)	Histol type

Toulmonde (114)^b^	586	64.5	76	66	46	–	–	Age (1.0–1.9) Male gender (1.3–2.3) Grade 3 (2.7–6.2) Adj organ invasion (1.2–2.2) Piecemeal res (1.3–3.0)	–	–	–	Male gender (1.1–2.0) Adj organ invasion (1.2–2.1) Surgeon specialization (0.4–0.7) Piecemeal res (1.9–4.5) Periop RT (0.4–0.7)	–

*^a^Includes primary and recurrent RPS*.

*^b^Prognostic factors calculated for a subgroup of patients (*N* = 389) who underwent complete resection*.

*NS, not statistically significant; RT, radiotherapy; ALT, atypical lipomatous tumor; res, resection; vs, versus; LiSa, liposarcoma; CoRes, complete resection; OS, overall survival; DSS, disease-specific survival; DFS, disease free survival; LRFS, local recurrence free survival. Numbers between brackets represent the 95% confidence interval of the hazard ratio or risk ratio*.

### Radiation therapy

Even after optimal resection of RLS, local recurrence remains common and constitutes the most frequent cause of death. Therefore, adjuvant radiation therapy (RT) may constitute a valuable treatment option in order to improve local control, specifically with involved margins or high-grade tumors. In patients with soft tissue sarcoma of the extremity, two small randomized trials have shown that postoperatiive external beam radiotherapy or brachytherapy improve local control, but do not benefit overall survival (115–117). In retroperitoneal soft tissue sarcoma, a myriad of small trials has been published, which show marked variation in RT dose, fractionation, concurrent use of chemotherapy, delivery method (external beam or brachytherapy), timing (preoperative, intraoperative, or postoperative), and energy carrier (photons, electrons, protons, or carbon ions) (118).

Preoperative RT is usually regarded as the treatment sequence of choice. First, preoperative radiation helps to avoid damage to radiation sensitive structures and organs, which usually fill in the resection bed after removal of these large tumors. Second, treatment compliance is usually better and related toxicity less in the preoperative setting. Also, the biological effects of RT are enhanced in undisturbed, well perfused, and oxygenated tissue.

Table 6 illustrates published data on the use of preoperative RT in patients with retroperitoneal sarcoma. Most are small, retrospective series describing different histologies and treatment methods. Local control seems, on average, somewhat better compared to surgery alone series. Although the small numbers preclude any robust conclusion, most authors did not find any benefit of adding either IOERT or postoperative BT to the external beam RT. Only one small study compared surgery alone with preoperative external beam and intraoperative RT followed by surgery, and found that the combined modality resulted in improved local control without any difference in overall survival (119). Several authors have scrutinized data from the surveillance, epidemiology, and end results (SEER) database in an attempt to define the role of adjuvant RT in RLS (Table 7). The results are difficult to compare due to differences in inclusion period, inclusion criteria, case mix, and analytical method. As a general finding, adjuvant radiotherapy either did not benefit survival or did so in a subgroup of stage I patients only. Of note, the large majority of patients treated with radiotherapy were administered this treatment in the postoperative period.

**Table 6:** **Selected clinical studies of preoperative radiotherapy for retroperitoneal (lipo)sarcoma**.

Author	*N*	% LiSa	Treatment regimen.	5 years LRFS	5 years DFS	5 years OS/DSS
Gieschen (120)	37	22	EBRT (45–50 Gy/1.8 Gy)/additional IOERT (10–20 Gy; *N* = 20)	60.6/83.3	25.6/68.2^a^	30/74.4^a^
Pawlik (121)	72	40	MDACC: chemoradiation (18–50.4 Gy with concurrent doxorubicin) and IOERT (*N* = 35)	60	46	50
			U Toronto: EBRT (45 Gy) and postop BT (25 Gy)			
Tzeng (122)	16	25	EBRT (45 + 57.5 Gy boost to volume at risk for positive margins)	80 @2 years	–	–
White (123)	27	50	EBRT 45–50 Gy after surgical tissue expander insertion	80	–	74
Caudle (124)	14	43	EBRT (45–50 Gy); additional IOERT (12.5–15 Gy, *N* = 5)	50 @2 years	–	74 @2 years
Ballo (125)	83^f^		EBRT (50 Gy); additional IOERT (15 Gy, *N* = 18), additional postop BT (*N* = 2)	40 @10 years	39 @10 years	44 @10 years
Yoon (126)	28^c^	50	EBRT (50 Gy); additional IOERT (10–12 Gy, *N* = 12)	90^d^,^e^	–	87^d^
Alford (127)	24	50	EBRT (45–50.4 Gy)	81.3	48.9	53.7
McBride (128)	33	48	EBRT (50 Gy); additional postop BT (77.5 Gy, *N* = 10)	–	45.4 @3 years	63.5 @3 years
Sweeting (129)	18	50	EBRT (45–50 Gy, *N* = 17); additional IOERT (12.5–20 Gy)	64	–	72
Smith (130)	40	70	EBRT (45–50 Gy/1.8 Gy)/additional postop BT (20 Gy; *N* = 19)	75/61	–	76^b^/52^b^
Stucky (119)	63	68	Surgery alone (*N* = 26)	46	–	60
			EBRT (45–50 Gy) and IOERT (10–20 Gy); *N* = 37	89^a^	–	60
Gronchi (131)	83	54	EBRT (45 Gy) with high dose ifosfamide; additional IOERT (10–12 Gy, *N* = 14)	–	44	59

OS, overall survival; DSS, disease-specific survival; DFS, disease free survival; LRFS, local recurrence free survival; EBRT, external beam radiotherapy; IOERT, intraoperative electron beam radiotherapy; BT, brachytherapy. MDACC, MD Anderson Cancer Center; U, University;

*^a^Statistically significant*.

*^b^Survival at 10 years*.

*^c^Includes eight patients who underwent postoperative RT*.

*^d^Survival at 3 years*.

*^e^In patients with primary tumors*.

*^f^Includes 33 patients who underwent postop RT; LiSa, liposarcoma*.

**Table 7:** **Studies based on data from the surveillance, epidemiology, and end results (SEER) database in an attempt to define the role of adjuvant radiotherapy in retroperitoneal sarcoma**.

Author	Inclusion	*N*	Inclusion criteria	Statistical methods	Significant covariates for OS/DSS	RT
Porter (132)	1973–2001	1226 surgery 428 surgery with RT (85.5% of RT postop)	Age ≥18	Logistic regression (use of RT)	–	Adjuvant radiotherapy use varies significantly with age, race, and geographical location
Nathan (133)	1988–2005	1365	Curative intent surgery	Cox regression	Age, sex, grade, histology	Unadjusted Cox analysis: HR for OS 0.78–1.15
Zhou (134)	1988–2005	1574	Age ≥18	Cox regression Stratified for AJCC stage	Surgery, age, sex, stage	Stage I: HR for OS 0.25–0.96 Stage II/III: HR for OS 0.58–1.06
Tseng (135)	1988–2004	1130 surgery 373 surgery with RT (80.4% of RT postop)	Age ≥18 Patients underwent surgery	Cox regression	Age, sex, histology, grade Complete resection	Cox regression: HR for OS 0.78–1.09 overall OS benefit in MFH (*P* = 0.002) and dedifferentiated liposarcoma (*P* = 0.08) in univariate analysis
Choi (136)	1988–2006	558 surgery 204 surgery with RT (80% of RT postop)	Age ≥20, single malignancy Curative intent surgery	Cox regression; propensity score matching	Age, sex, grade, stage	Cox regression: HR for DSS 0.87–1.56 After PS matching: no difference in DSS (*P* = 0.35) or OS (*P* = 0.1)

*OS, overall survival; DSS, disease-specific survival; HR, hazard ratio; MFH, malignant fibrous hstiocytoma; PS, propensity score*.

To date, no randomized trials have been completed or published comparing surgery alone with combined surgery and RT. The American College of Surgeons Oncology Group (ACOSOG) initiated a trial (Z9031) in 2004 comparing surgery alone versus fractionated RT followed by surgery. The primary endpoint was progression free survival at 5 years. This trial was terminated due to poor accrual. The European Organization for Research and Treatment of Cancer (EORTC) protocol 62092, which started in 2012, randomizes patients to either en-bloc surgery alone versus fractionated RT (50.4 Gy in 28 fractions) followed by en-bloc surgery. The primary endpoint is abdominal recurrence free survival. With a planned sample size of *N* = 256, completion of the inclusion period will take at least until 2019.

### Systemic therapy

Chemotherapy has an established role in the palliative management of advanced or metastatic soft tissue sarcoma. Active agents include the anthracyclines (doxorubicin and epirubicin) and the alkylating agent ifosfamide (137). In patients with resistant disease, gemcitabine, docetaxel, trabectedin, and pazopanib were established as effective second or third line options over the last decade (138). STS are a very heterogeneous group, and chemosensitivity is determined by histological type and grade. Examples include the response of angiosarcomas to paclitaxel and pegylated liposomal doxorubicin, the response of leiomyosarcomas to gemcitabine and docetaxel, and the response of desmoid tumors to liposomal doxorubicin (138). Similarly, the response of liposarcoma to chemotherapy differs according to histological subtype and grade. Investigators from the Royal Marsden Hospital investigated response to chemotherapy in 88 patients with liposarcoma (43% located in the RP) (139). They found a significantly higher response rate in myxoid liposarcoma compared to all other liposarcomas (48 versus 18%, *P* = 0.012). The response rate was 25% in dedifferentiated liposarcoma, while none of the well-differentiated liposarcomas responded. Also, response was significantly better in patients with liposarcoma of the upper limb (75%) or lower limb (36%) compared to other locations (18%). Italiano and coworkers reported the role of chemotherapy in unresectable and/or metastatic well-differentiated and dedifferentiated liposarcoma (77.5% retroperitoneal) based on retrospective analysis of data from 10 centers (139). Seventy-three percent of the included 208 patients had at least one metastatic site. Using RECIST criteria, response was complete in 1% and partial in 11%, while stable disease and progression were seen in 48 and 39%, respectively. No difference was observed in response rate between WD and DD liposarcoma.

Because well-differentiated liposarcoma and dedifferentiated liposarcoma respond poorly to systemic chemotherapy, it is essential that novel molecular targets will be identified to provide new possibilities for therapies. The reported results of recent clinical trials for novel systemic therapies in advanced liposarcoma are overall encouraging. In the past decade, results from clinical trials have identified several novel systemic therapies in soft tissue sarcoma, many of which have potential efficacy in liposarcoma (5, 140). In contrast to conventional cytotoxic chemotherapies, which are non-specific, the majority of these novel therapies are based on the understanding of disease biology inherent to a given sarcoma histology, in many cases targeting a specific, aberrant genetic, or molecular pathway. For the majority of novel therapies, treatment efficacy is heavily dependent on subtype. Reported human studies and clinical trials for novel systemic therapies in liposarcoma are summarized in Table 8. Targeting *MDM2* or *CDK4* (*MDM2* and *CDK4* antagonists) in well-differentiated liposarcoma and dedifferentiated liposarcoma has been of interest for several years (5, 140–143). Based on the fact that well-differentiated liposarcoma and dedifferentiated liposarcoma are relatively resistant to systemic chemotherapy, *MDM2* and *CDK4* targeted therapy may be a very promising approach, especially for advanced or unresectable well-differentiated and dedifferentiated liposarcoma. A class of imidazoline compounds, termed nutlins, has been identified as potent and selective small-molecule *MDM2* inhibitors. RG7112 (Hoffmann-La Roche) (Phase I, neoadjuvant) is a member of the nutlin family and is the first *MDM2* antagonist to be assessed clinically. RG7112 is a potent inhibitor of p53-MDM2 binding that effectively stabilizes p53 protein, activates p53 signaling, and inhibits cancer cell growth (140, 141). Flavopiridol-*CDK4* inhibitor and PD0332991-*CDK4*/*CDK6* inhibitor (Pfizer) (Phase I) are potent *CDK4* inhibitors, preventing downstream phosphorylation of the retinoblastoma (RB) protein. *CDK4* inhibition would thus restore native cell cycle regulation and prevent uncontrolled tumor cell proliferation (140, 143). Several other interesting candidate targets for novel systemic therapies, including *YEATS4*, *c-jun*, *JNK*, and others, have been reported but have not yet been tested, to our knowledge, in the setting of a clinical trial (140, 144). PPAR are critical regulators of normal adipocyte differentiation. PPAR gamma is one of the three isoforms that forms a heterodimeric complex with the retinoid X receptor to regulate transcription of adipocyte-specific genes involved in the terminal adipocyte pathway. Activation of PPAR gamma by PPAR gamma agonists (troglitazone, rosiglitazone, efatutazone; phase I and II) represents an attractive target, particularly in dedifferentiated liposarcoma, myxoid/round cell liposarcoma and pleomorphic liposarcoma, as a mechanism to revert these subtypes to a well differentiated phenotype with potentially more indolent disease progression (5).

**Table 8:** **Molecular therapeutic targets and agents in soft tissue sarcoma**.

	Molecule	Target/mechanism	Liposarcoma histologic subtype	Clinical phase
Marine derived compounds	Trabectedin (145–147)	Binding of DNA minor groove; direct interaction with FUS-CHOP fusion protein	Myxoid/round cell liposarcoma	Phase II, retrospective and neoadjuvant
	Eribulin (148)	Microtubule inhibitor	Dedifferentiated liposarcoma	Phase II
MDM2 antagonists	RG7112 (143, 149)	p53-MDM2 inhibitor	Well-differentiated and dedifferentiated liposarcoma	Phase I (neoadjuvant)
	RG7388 (150)	p53-MDM2 inhibitor	Well-differentiated and dedifferentiated liposarcoma	Phase I
CDK4 antagonists	Flavopiridol (151)	Pan-CDK inhibitor, including CDK4	Well-differentiated and dedifferentiated liposarcoma	Phase I
	PD 0332991 (142)	CDK4/6 inhibitor	Well-differentiated and dedifferentiated liposarcoma	Phase I
Other	Troglitazone (152) Rosiglitazone (153) Efatutazone (154)	PPAR gamma agonist	All liposarcoma types	Phase I, II
	Nelfinavir (155, 156)	SREBP-1 inhibitor	Well-differentiated and dedifferentiated liposarcoma	Phase I
	Sunitinib (157)	Tyrosine kinase receptor inhibitor	All liposarcoma types	Phase II
	Panobinostat (158)	Histone deacetylase inhibitor	All liposarcoma types	Phase II

Tyrosine kinase receptors are a diverse family of surface molecules recognized for their critical role in regulating multiple aspects of carcinogenesis, tumor cell proliferation, and disease progression (e.g., angiogenesis, metastasis) across many solid tumor types. In the presence of a specific growth factor ligand, tyrosine kinase receptors and their associated downstream molecules are frequently over-expressed or mutated, leading to constitutive activation or aberrant signaling. In a single institution study of 48 patients at the Moffitt Cancer Center, Tariq Mahmood et al. reported impressive efficacy with sunitinib, especially for liposarcoma (157). It is unclear why sunitinib, but not pazopanib or sorafenib, has anti-tumor activity in liposarcoma despite having similar molecular targets (159, 160).

## Conclusion

Retroperitoneal liposarcoma is a rare tumor, and exhibits considerable histological heterogeneity. Adequate staging and grading is essential in guiding the therapeutic approach. Macroscopically complete resection offers the best chance of prolonged recurrence free survival. The value of extensive, “compartmental” resection in these patients is at present not well defined, but influenced by disease biology. Preoperative radiotherapy may lower the risk of locally recurrent disease, and a prospective, randomized EORTC study is currently open for inclusion. In parallel with the unraveling of the molecular pathways underlying sarcoma genesis, several targeted agents are in active development that may contribute to the available systemic treatment options in the near future.

## Conflict of Interest Statement

The authors declare that the research was conducted in the absence of any commercial or financial relationships that could be construed as a potential conflict of interest.
